# Climate Change Disproportionately Increases Herbivore over Plant or Parasitoid Biomass

**DOI:** 10.1371/journal.pone.0040557

**Published:** 2012-07-18

**Authors:** Claudio de Sassi, Jason M. Tylianakis

**Affiliations:** School of Biological Sciences, University of Canterbury, Christchurch, New Zealand; Duke University, United States of America

## Abstract

All living organisms are linked through trophic relationships with resources and consumers, the balance of which determines overall ecosystem stability and functioning. Ecological research has identified a multitude of mechanisms that contribute to this balance, but ecologists are now challenged with predicting responses to global environmental changes. Despite a wealth of studies highlighting likely outcomes for specific mechanisms and subsets of a system (e.g., plants, plant-herbivore or predator-prey interactions), studies comparing overall effects of changes at multiple trophic levels are rare. We used a combination of experiments in a grassland system to test how biomass at the plant, herbivore and natural enemy (parasitoid) levels responds to the interactive effects of two key global change drivers: warming and nitrogen deposition. We found that higher temperatures and elevated nitrogen generated a multitrophic community that was increasingly dominated by herbivores. Moreover, we found synergistic effects of the drivers on biomass, which differed across trophic levels. Both absolute and relative biomass of herbivores increased disproportionately to that of plants and, in particular, parasitoids, which did not show any significant response to the treatments. Reduced parasitism rates mirrored the profound biomass changes in the system. These findings carry important implications for the response of biota to environmental changes; reduced top-down regulation is likely to coincide with an increase in herbivory, which in turn is likely to cascade to other fundamental ecosystem processes. Our findings also provide multitrophic data to support the general concern of increasing herbivore pest outbreaks in a warmer world.

## Introduction

Global environmental changes affect all living organisms, with complex consequences for biodiversity, ecosystem structure and function [1], [2]. Predicting generalities in the direction of such changes represents one of the major challenges in ecology. However, the complexity of this task is exacerbated by the great variability of responses observed, across biomes, space, time, and scales of biotic organization [3]. Climate has effects at all levels of organization, from population dynamics to community composition and species-specific responses [4], [5], and it has strong impacts on ecosystems and their services [1], [6], [7]. A wealth of studies have shown that climate warming, provided it is not too extreme, generally increases plant net primary production [8]. However, warming has also been shown to have positive effects on herbivore population size and herbivory [9], which may counteract the increased plant growth. Furthermore, the net effect of climate on herbivores will result both from direct and plant-mediated effects and from top-down control by natural enemies, and this complexity may be partly responsible for the highly-variable responses of herbivores to different environmental change drivers [5].

The net ecosystem balance arising from the combination of these effects therefore depends on the relative response of individual trophic levels. A vast body of literature has addressed the effect of climate on plant-herbivore and prey-predator systems, but it disproportionately represents studies looking at pairs of interacting species, rather than larger modules or communities at once [3], [5]. Despite the insights on specific mechanisms (e.g., phenological mismatches, shifts in competition, prey defense and palatability) gained from this approach, such studies do not allow generalizations to be made on the *relative* impact of climate or other change drivers at different trophic levels. In fact, only a handful of investigations have specifically considered overall responses at different trophic levels. For example, Voigt and colleagues focused on covariance in the response to multiple climatic factors of community composition, at different trophic levels [10] and functional groups [11], and concluded that sensitivity (i.e population fluctuations) to climate increases with trophic level. Focusing on a model system including a raptor bird, four passerine species and two caterpillar species, Both *et al.* [12] showed that the response of consumers is weaker than that of their resource. However, this result is contrasted by a recent study showing a climate-induced increase in synchrony between food demand and availability in a similar caterpillar-passerine system [13]. This latter result indicates that variability in species responses may not necessarily match on the overarching community-wide response. Nevertheless, these results imply that climate change is likely to prompt changes in the trophic structure of communities, which could directly or indirectly affect ecosystem processes such as nutrient cycling, herbivory and predation [14], [15].

Finally, in addition to indirect effects on species through changes at adjacent trophic levels, organismal responses to climate could be altered by co-occurring changes in the biotic and abiotic environment, such that recent literature has called for the integration of multiple drivers in global change research [5], [16].

For example, biologically-available nitrogen deposition in non-agricultural systems has increased rapidly and become a major driver of biotic change [17]. As well as generally increasing net primary productivity (NPP), nitrogen has been shown to alter plant competitive interactions [5], [18], [19] and drive biodiversity losses [20], [21]; effects that can percolate to higher trophic levels [22]. Despite the logical assumption that nitrogen will, in contrast to temperature, only affect herbivores via bottom-up effects [5], the interaction of the direct effect of temperature with changes in basal resource availability triggered by nitrogen, create a complex interplay that shows more context dependence than either effect in isolation [23], [24]. Finally, the combined impact of warming and nitrogen on natural enemies is largely unknown, though N deposition tends to benefit predators [5], while climate warming can destabilize predator-prey interactions [25]. Thus, the interactive effects of temperature and N on plant growth [26] and herbivores [5], complicated by the general absence of data on their effect on natural enemies, suggest that these drivers may have complex, non-additive effects on trophic balance.

In this study, we examine how biomass at three trophic levels (plants, lepidopteran herbivores and their parasitoids) responds to co-occurring increases in temperature and nitrogen. We use seminatural grasslands as a model system, due to their global ubiquity [27] and importance for grazing agriculture. Furthermore, they are known to respond to N addition [20], and more strongly to warming than to other climate drivers such as CO_2_ concentration and drought [28].

We use a field experiment along an altitudinal gradient, combined with an artificial warming experiment under controlled field conditions, and measure how total biomass of plants, herbivores and parasitoids, as well as parasitism rates, respond to elevated temperature and nitrogen treatments.

## Materials and Methods

### Study Site: Altitudinal Gradient Experiment

We established our experiment near Lewis Pass, North Canterbury, New Zealand (Appendix S1). The valley is located at the foothills of the Southern Alps, and ranges from 600 to 1,700 m elevation. The climate is cool and humid, with a mean annual rainfall of 1560 mm and a mean annual temperature of 9.1°C [29]. The wider experimental area is characterized by montane tussock grassland, dominated by native species in the genus *Festuca, Poa, Rytidosperma*, and *Chionochloa* at higher altitudes. These species are typical of semi-arid to humid, montane and subalpine zones in New Zealand [30]. The inter-tussock ground is generally dominated by stock-palatable Eurasian species (particularly *Agrostis capillaris, Anthoxanthum odoratum, Trifolium* spp.), which were over-sown after forest clearing in the late 1800s. At present, the area is farmed at very low intensity, with a stock density of less than 1 sheep per hectare, and no nitrogen fertilizer is applied.

### Experimental Design and Sampling of Altitudinal Gradient

**Figure 1:**
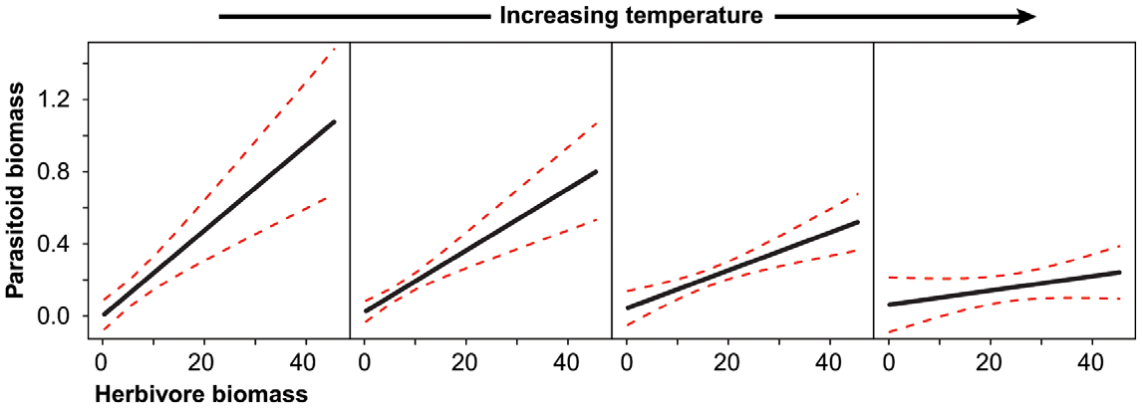
The correlation between herbivore biomass and parasitoid biomass along the temperature gradient. Panels are ordered from left to right (increasing temperature). Thus, the left plot represents the herbivore-parasitoid biomass relationship at the lowest temperature and the right plot is for the highest temperature. Black lines represent fitted values from out mixed effects model, dashed lines show the upper and lower range of the standard error. This plot highlights the decreasing slope of biomass correlation with increasing temperature.

To generate a climatic gradient, we used an elevation gradient as a ‘space for time substitution’ [31], [32]. We established five vertical transects of three plots, each at 150 m intervals of elevation, such that there was a total of 300 m difference in altitude between the lowest and the highest plot in each transect. Transects were at least 600 m apart (twice the vertical length of each individual transect, see Table 1 and Figure 1 in Appendix S1).

**Table 1:** Elevational gradient experiment: coefficient table for the combined effect of the drivers on A) plant biomass and the effect of the drivers and resource (plant or herbivore host respectively) biomass on total biomass of B) herbivores and C) parasitoids.

A) Plants							
	Values	Std. Error	df	t-value		P-value	
(Intercept)	9830.12	10020.47	13	0.98		0.345	
Temperature	−232.27	1873.78	9	−0.12		0.904	
Nitrogen	10577.54	10907.60	13	0.97		0.350	
Temperature : nitrogen	−1408.00	2042.77	13	−0.69		0.503	
**B) Herbivores**							
(Intercept)	−43.10	10.53	12	−4.09		0.002	**
Plant biomass	0.0005	0.0002	12	2.37		0.036	*
Temperature	9.32	1.94	9	4.80		0.001	**
Nitrogen	−10.87	13.60	12	−0.80		0.440	
Temperature : nitrogen	2.43	2.53	12	0.96		0.355	
**C) Parasitoids**							
(Intercept)	0.07	0.18	11	0.38	0.713		
Herbivore biomass	0.051	0.01	11	4.41	0.001		**
Temperature	−0.003	0.03	9	−0.07	0.943		
Nitrogen	−0.29	0.20	11	−1.45	0.174		
Herbivore biomass : warming	−0.007	0.002	11	−3.72	0.003		**
Temperature : nitrogen	0.04	0.04	11	1.26	0.230		

Asterisks indicate level of significance (. ≤0.1, * ≤0.05, ** ≤0.01).

All plots had a similar incline and vegetation type, and faced north or north-west. Note, however, that analyses incorporated transect as a random (blocking) factor, so any environmental differences among transects would not confound treatment effects. To maintain similar characteristics, transects were not all positioned at exactly the same elevation, so plots ranged from 650 m at the lowest point to 1073 m a.s.l at the highest (423 m of total elevation span). This provided a total temperature gradient of 2.83°C across all plots (the average temperature in each plot over the entire period of data recording ranged from 3.89 to 6.72°C). This temperature gradient falls within the range of temperature increases predicted for the region within the next 100 years [33] and provided a better fit to the data than using changes in extreme temperatures (maxima or minima) as predictors (data not shown). Similarly, we found a strong correlation (R^2^>0.91) between yearly mean temperature and the mean temperature of the growing season (September to January). Mean growing season temperature did not increase the fit of the data and we therefore used the annual mean for consistency with our sampling regime.

Local topography may create significant microclimatic variation, which could modify temperature over short vertical distances that override the more general altitudinal trends [34]. This allowed us to test the effects of temperature *per se*, partially uncoupled from the effects of other environmental variables that co-vary with elevation (such as oxygen availability and radiation [32]. Temperature was recorded in each plot using Hobo series ProV2 data loggers, protected by a sun shield, logging temperature at 1 h intervals from February to December 2009. We used the overall mean site temperature for this period as a predictor variable in the analysis.

At each elevation, we established a 24×12 m sampling plot. We further subdivided each plot into two 12×12 m subplots, and randomly assigned one of these to a nitrogen addition treatment (addition or control with no added N). This resulted in a split-plot design, with temperature varying at the scale of plots (n = 15), blocked by transects (n = 5), and N treatments applied to subplots (n = 30) nested within plots. The N fertilization treatment comprised a total application of 50 Kg ha^−1^ yr^−1^, which falls within the current range of globally-observed rates of atmospheric deposition [35]. Precise N deposition rates for the study region are not known, but expansion of dairy farming across New Zealand is driving rapid increases in N fertilizer application [36], which will likely impact adjacent semi-natural grasslands. Nitrogen fertilizer was applied in the form of Calcium Ammonium Nitrate (CAN) granules (Ravensdown LTD, New Zealand). This form of fertilizer combines fast and slower release of biologically-available nitrogen, and has been used previously to simulate atmospheric deposition [21].

We began N addition in September 2008, by adding 40% of the total year budget (20 Kg ha^−1^ yr^−1^, 1066 g CAN per subplot) and applying the remaining 60% in 4 pulses, evenly distributed over the next 12 months, by sprinkling the dry granules throughout the treated subplot. Fertiliser addition continued at a rate of 50 Kg ha^−1^yr^−1^ until sampling was completed in December 2009.

Although initial sampling of insects began in October 2008, here we present data only from samples where biomass was measured, which were those collected from May to December 2009, i.e., approximately a year after starting the nitrogen fertilization treatment. To minimize disturbance and depletion of caterpillars in the experimental area, we subdivided each 12 x 12 m subplot into 4 strips of 3×12 m each, and sequentially sampled one strip only during each sampling round. This allowed monthly sampling, but ensured a time window of at least 4 months before re-sampling of the same section. This timeframe is substantially longer than the average larval life stage of Lepidoptera in our study area, and therefore prevented bias in the abundance of any sample caused by depletion from previous sampling rounds.

We searched all the tussocks within the 3×12 m strip at each sampling round. Plant searches involved thorough teasing apart of denser vegetation to locate any hidden larvae.

### Study Site: Artificial Warming Experiment

**Figure 2:**
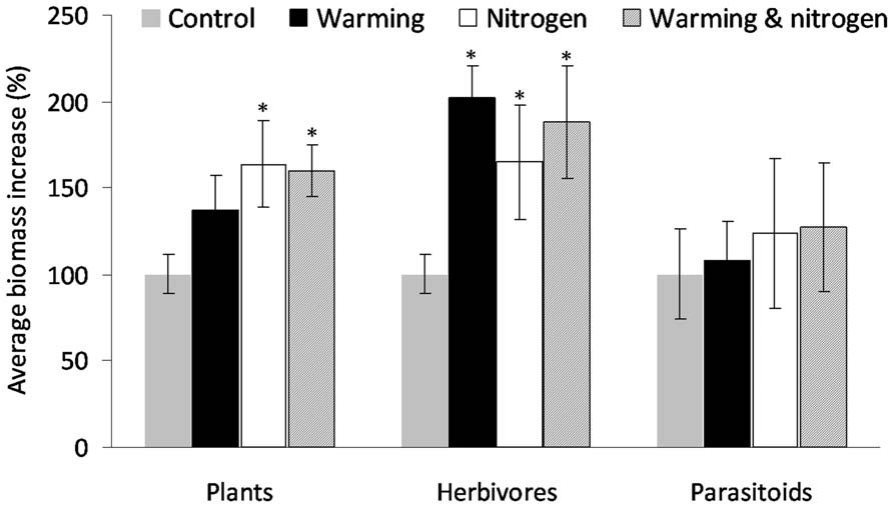
The effect of the global change drivers (warming, nitrogen and their combination) on the percentage increase in biomass relative to the control treatment for plants, herbivores and parasitoids. As the percentage increase is scaled within trophic levels, this graph allows a direct comparison of the effect of the drivers within and across trophic levels (plant, herbivores and parasitoids). Asterisks depict significant differences relative to the group control.

We set up an artificial warming experiment adjacent to the University of Canterbury field station at Cass in the Waimakariri River catchment, South Island of New Zealand (Figure 2 in Appendix S2). The Cass field station lays at 640 m a.s.l., a mean annual rainfall of 1300 mm (1918-1965) is uniformly distributed throughout the year, and typical monthly mean air temperatures range from 1.6°C (July) to 15.7°C (February). Snow lies for some days each winter (June-September). The climate of the area is described in detail by Greenland [37].

The study area is embedded in a montane short-tussock grassland with very similar characteristics to the environment of the altitudinal gradient experiment, although the two experimental locations are over 60 Km apart and belong to different catchments. The intertussock area is dominated by Eurasian grasses oversown for pastoral purposes. The area surrounding the Field Station shows a strong dominance of *Agrostis capillaris* and *Anthoxanthum odoratum* [38].

### Experimental Design and Sampling of Warming Experiment

The experiment comprised a 2×2 factorial design, with warming and nitrogen as treatments with two levels each (control vs. elevated) and five true replicates per treatment combination, totaling 20 plots. We dug a 24 m by 19 m experimental area in October 2008, to a depth of 20 cm to establish the 20 3.5×3.5 m plots (12.25 m^2^), each separated by a 1 m corridor. We then leveled the ground and installed custom-made electric heating cables (Argus Heating Ltd, Christchurch, New Zealand: coiled copper wire on fiberglass core and silicon coating) in half of the plots, and dummy cables in the remaining (unheated) plots. Each plot was fitted with two coils of 45 m meters each, resulting in a spacing of 14 cm between cable lines. Heating power totals 940 Watts per plot or a power density of 76W/m^2^. Similar power output has been recommended [39] and successfully used in previous underground heating experiments [40].

In each warmed plot, we installed three thermocouples (Type E, Chromel-Constantan, Campbell Scientific, USA) at 10 cm depth and standardized position relative to the heating cables (1 directly above the cable, 1 between two heating cables, 1 between the other two thermocouples), to capture any potential temperature differences within the plot driven by distance from the heating cables. In each control (unheated) plot, we installed 1 thermocouple at the same depth. The thermocouple in the control plot provided a baseline measure of ambient temperature so that the warmed plots could be kept at a constant temperature above ambient.

We homogenized all the extracted soil by mixing it with a digger to remove any confounding nutrient or bio-geochemical gradient before re-installing it in the experimental area and leveling the ground to ensure constant ground depth relative to the cables We planted well established (at least 3 month old) individuals of four species of tussock grasses, which were common to the general area and also present in the altitudinal gradient experiment (50× *Poa cita,* 50× *Festuca novae-zelandiae,* 12× *Chionochloa rigida* and 12× *Chionochloa flavecens* per plot), in a consistent composition and layout for each plot). This resulted in each plot being planted with 144 individual plants, amounting to 2880 tussocks in total. We completed the set up and planting in January 2009 (see Figure 3 in Appendix S2). To minimize water stress to the recently-planted tussocks in the height of the first summer, we installed an automated watering system, which ran for half an hour at dawn and after sunset until May 2009. We first activated the warming treatment in April 2009, after the plants had established for over three months. However, this required adjustment and tuning of temperature differences, and the experiment was fully operational by late June 2009. We paired each warming plot with its spatially-closest control plot to keep the warmed treatments at 3°C above ambient, logging the temperature of all thermocouples every minute using two Campbell CR1000 (Campbell Scientific, USA) data loggers. The average temperature of the thermocouples in the warming plots is used against the control plot to switch the power on and off as required (See Figures 4 and 5 in Appendix S2 for details on the temperature control). The three degrees of warming achieved in this experiment is in line with the temperature gradient we found in the field experiment and with the predictions of global (and New Zealand) warming scenarios for the next 100 years [33].

The nitrogen treatment application, using the same fertilizer as the gradient experiment, began shortly after planting, by adding the equivalent of 25 Kg ha^−1^ yr^−1^ in late January 2009. Applications reached a total of 50 Kg ha^−1^ yr^−1^ with three evenly-distributed applications during the rest of the year. Fertilization treatments continued in 2010 with five applications of 10 Kg ha^−1^ yr^−1^, one every two months except the winter months of July and August, where the plots were often covered in snow. The decision to use five applications arose from a tradeoff between maximizing frequency of applications (to resemble natural deposition), yet applying enough to practically allow even application across the entire treated plot.

We began sampling insects in January 2010, that is, a full year after plot establishment and planting. Sampling continued at monthly intervals until June 2010 (i.e. mid winter, when snow cover made sampling impractical), and resumed at monthly intervals from September to December 2010, totaling 11 sampling rounds. To minimize disturbance and depletion of caterpillars in the experimental area, we sampled half of each plot during each round, alternating between the two halves. This ensured a time window of at least 8 weeks before re-sampling of the same section. Sampling entailed visually searching for caterpillars on tussock plants, teasing apart the dense vegetation to find any hidden larvae.

Both the artificial and the gradient experiment present a number of caveats in their design: using natural-gradient studies has limitations in the ability to explain the response of communities to temperature changes, as populations may already have adapted to the different conditions [32]. Additionally, changes to mean temperatures following global warming may be strongly influenced by changes to frequency and magnitude of extreme temperature events [33], which remain unaccounted for in our study. Similarly, artificial warming experiments such as the one presented in this study can be criticized for the necessarily small scale, and limitations of any heating method used in simulating global change [41]. However, most experiments to date have used one of these methods. In this study, we used both a large-scale field experiment combined with a manipulative controlled field experiment, finding largely consistent results that provide a good degree of confidence that the patterns found were due to the generalities of communities’ response to simulated global-change drivers, rather than spurious effects of any particular experimental approach.

### Insect Rearing

For both experiments, we identified each individual larva to morphospecies. To allow collection of parasitoids, we individually reared all larvae to maturity (emergence of the adult moth or parasitoid) in a climate-controlled room, with a constant temperature of 16 degrees, relative humidity of 60% and a light cycle of 16L:8D. The feeding protocols varied according to the species requirements. All parasitoids were identified to species level where possible, and to morphospecies for organisms lacking a recognized classification. We sought the expertise of two taxonomists to help with the identification: John S. Dugdale developed a larval key for Lepidoptera and confirmed the identity of all the tachinid flies, and Jo Berry validated hymenopteran morphospecies and formally identified all known species. The individual rearing of every herbivore larva allowed us to estimate the rate of parasitism (proportion of larvae from which a parasitoid emerged).

Larvae that died during rearing (22% in the altitudinal gradient and 42% in the warming experiment) were excluded from all analyses. A total of 4224 caterpillars (39 species) and 860 parasitoids (41 species) were included for the altitudinal gradient experiment, whilst the artificial warming experiment comprised 893 caterpillars (26 species) and 331 parasitoids (21 species).

### Biomass Measurements

To estimate effects of temperature and N on larval biomass, we weighed the caterpillars (Mettler Toledo analytical balance accurate to 0.0001 g) directly after collection for all samples. We estimated total herbivore biomass as the sum of the larval weight of all individuals in each plot. As we could not always observe parasitoids as soon as they emerged, there is a risk that individual parasitoid weight could be biased by the time between emergence and being discovered. Furthermore, unlike herbivore mass, which was measured directly after collection, parasitoid body mass can only be measured at emergence, and could therefore be strongly determined by the age at which the host larva was brought into the laboratory for rearing, and the food provided to the growing larva. Therefore, to avoid the possibility that these effects could generate spurious differences across treatments, we calculated the total parasitoid biomass for a plot by multiplying the total counts of each species by the average weight of that species. We obtained each species average by weighing 20 adult individuals of each species, or all individuals for the rarer species (less than 20 individuals).

To estimate plant biomass without disruptive sampling of the plots, we estimated the total tussock volume in each plot. To obtain the total tussock volume, we first calculated the mean tussock volume per plot by measuring a subset of randomly-selected tussocks (20 in the warming experiment, 30 in the gradient experiment. We measured basal circumference and height from the ground to the highest leaf, and then calculated the cylinder volume [42]. After obtaining the average tussock volume for each plot, we multiplied it by the total count of tussock individuals. To convert plant volume to biomass, we measured the volume of 10 tussock plants from our glasshouse cultures following the same procedure as above. We then clipped them to ground level and dried the leaf material at 60°C for 48 hours. We used a linear regression to test how well volume approximated dry weight, and found a significant relationship (F_1,8_ = 20.68, P = 0.001, R^2^ = 0.72).

### Data Analysis

We carried out all analyses using R version 2.12.0 (2010 The R Foundation for Statistical Computing). To account for our split-plot design in the gradient experiment, we used general linear mixed effects models [43], within the nlme package [44]. We used total (summed) plant, herbivore or parasitoid biomass as the response variable, with a Gaussian error distribution. We included nitrogen treatment as a fixed factor and temperature as a (fixed) variate, with plots nested in transects as random effects. Biomass of consumer trophic levels is likely to be highly correlated with the biomass of the trophic level below (its resource). Therefore, we included the biomass of plants as a variate in the model predicting herbivore biomass, and herbivore biomass in the model for parasitoids.

We initially included all possible interactions, then simplified the model by removing non-significant interaction sequentially, each time assessing changes in Akaike Information Criterion (AIC) scores before any further simplification. This allowed us to determine if the effects of the drivers on consumer biomass persisted after accounting for variation explained by resource biomass, i.e. if there was any direct effect of the drivers beyond the bottom-up, resource-biomass-mediated effects. To highlight the differential response between trophic groups, we calculated an herbivore to plant biomass ratio and a parasitoid to herbivore biomass ratio. We used a logit transformation for these ratios to meet the assumptions of normality, then tested them each as a response variable in a mixed effects model with the two global change drivers as a predictor and a Gaussian error distribution.

In addition to biomass changes, the activity of natural enemies may respond to the treatments (e.g., higher activity due to higher metabolic rates with increasing temperature, or altered attack rates as host quality changes under elevated N). Because such a response may not have been captured by looking solely at changes in biomass, we tested the response of overall parasitism rates to the drivers. We modeled parasitism rates using a generalized linear mixed model with a binomial error distribution, carried out in the lme4 package [45]. The proportion of all herbivores that were parasitised was the response variable, and the drivers temperature and nitrogen as predictors.

To test for changes in biomass at each trophic level in the artificial warming experiment, given the full factorial design, we used general linear models (the lm function in the base package of R). We used total (summed) plant, herbivore or parasitoid biomass as the response variable, with temperature and nitrogen as fixed factors. We followed the same procedure as in the altitudinal gradient experiment by including resource biomass (biomass of plants as a variate in the model predicting herbivore biomass, and herbivore biomass in the model for parasitoids) alongside the drivers, including all interactions and subsequently simplifying the model as above. To highlight changes in total biomass within each trophic level, we also tested the relative percentage increase in biomass (arcsine square root transformed to meet the assumptions of normality and homoscedasticity) compared with the control treatment for each trophic level. We did not carry out this analysis in the gradient experiment because the use of temperature as a variate rather than the categorical (warming vs control) used here did not allow an equally effective comparison. We tested biomass ratio using the same procedure as in the altitudinal gradient experiment. To test the response of parasitism rates to the driver treatments, we used binomial errors and a logit link function in the glm function of the base package in R.

## Results

### Altitudinal Gradient Experiment

In the gradient experiment, we found no effect of the drivers on plant biomass (Table 1A). Herbivore biomass was positively correlated with plant biomass, but a strong effect of temperature on herbivores remained even after controlling for resource biomass (Table 1B). In contrast, a trend towards a positive effect of nitrogen on herbivore biomass (*t* = 1.82, d.f = 9, *P* = 0.090) disappeared when plant biomass was included in the model. Parasitoid biomass was positively correlated with host resource biomass (Table 1C), but did not respond directly to the treatments. Interestingly, we found a negative interaction between herbivore biomass and temperature, such that the positive relationship between herbivore biomass and parasitoid biomass was significantly weaker at higher temperatures (Table 1C, Figure 1).

We also found that increasing temperature led to an overall increase in the biomass ratio of herbivores to plants (*t* = 3.66, d.f. = 9, *P* = 0.005) and a tendency for a decrease in the ratio of parasitoid biomass to herbivore biomass (t = −1.91, d.f. = 9, P = 0.084, see Table 1 in Appendix S3). Concordantly, we found a negative effect of both drivers on parasitism rates, with a significant interaction such that the drivers acted sub-additively (Temperature: Z = −2.15, P = 0.031, Nitrogen: Z = −2.11, P = 0.034, interaction: Z = 1.98, P = 0.047).

### Warming Experiment

In the warming experiment, relative biomass responses to each driver differed across the different trophic levels (Table 2, Figure 2). There was no significant relative change in plant biomass at high temperature, but there was a significant increase in the nitrogen treatment (both in the absolute biomass, Table 2A, and in the mean (± SE) percent change relative to control  =  +63.8% ±24.9, P = 0.016), which remained when temperature and nitrogen were combined (+59.9% ± SE 15.2, non-significant warming x nitrogen interaction: Table 2A). In contrast, herbivore biomass on average doubled in response to temperature (relative change of +102% ±18.6, P = 0.006, for absolute change in total biomass see Table 2B) and was marginally higher in the nitrogen treatment (+64.7± SE 32.9, P = 0.062), with combined treatments showing a weakly sub-additive effect (+88.1% ± SE 33.1, P = 0.095). Herbivore total biomass was positively correlated with plant biomass but, nevertheless, retained a positive effect of temperature (Table 2B), consistent with the altitudinal gradient experiment. In contrast, the marginally-significant (P = 0.062) main effect of nitrogen on herbivores disappeared after plants were included in the model, providing some evidence that nitrogen effects on herbivores were indeed bottom-up.

**Table 2:** Artificial warming experiment: coefficient table for the combined effect of the drivers on A) plant biomass, and the effect of the drivers and resource (plant or herbivore host respectively) biomass on total biomass of B) herbivores and C) parasitoids.

A) Plants						
	Value	Std.Error	df	t-value	P-value	
(Intercept)	2036.54	377.49	12	5.3949	<0.001	**
Warming	751.10	480.56	12	1.5630	0.144	
Nitrogen	1299.46	480.56	12	2.7040	0.019	*
Warming : nitrogen	−828.69	679.62	12	−1.2193	0.246	
**B) Herbivores**						
(Intercept)	−0.18	1.37	15	0.13	0.898	
Plant biomass	0.002	0.0005	15	3.68	0.002	**
Warming	2.84	1.22	15	2.33	0.034	*
Nitrogen	0.15	1.34	15	0.16	0.910	
Warming : nitrogen	−1.70	1.69	15	−1.01	0.330	
**C) Parasitoids**						
(Intercept)	0.03	0.04	15	0.62	0.548	
Herbivore biomass	0.02	0.007	15	2.84	0.012	*
Warming	−0.08	0.05	15	−1.81	0.098	**.**
Nitrogen	−0.03	0.04	15	−0.60	0.556	
Warming : nitrogen	0.06	0.06	15	0.95	0.358	

Asterisks indicate level of significance (. ≤0.1, * ≤0.05, ** ≤0.01, *** ≤0.001).

Finally, parasitoid relative biomass did not differ from control under any treatment combination (P>0.1 in all cases). After including herbivore biomass alongside the treatments predicting total parasitoid biomass, we found a positive correlation between resource and consumer biomass and only a trend (*P*<0.1) towards a negative effect of temperature on parasitoid biomass after controlling for the effect of herbivore biomass (Table 2C).

Overall, the observed changes in biomass at higher temperatures led to an increase in the biomass ratio between herbivores and plants (t = 2.50, P = 0.023) and a tendency towards a decrease in the ratio of parasitoid to herbivore biomass (t = −1.78, P = 0.093, see Table 1 in Appendix S3). In contrast, we found no effect of the drivers on parasitism rates in the warming experiment.

## Discussion

We found distinct responses of biomass at different trophic levels under elevated temperature and nitrogen and, overall, these results were consistent between two experiments that strongly differed in spatial scale and design. In particular, herbivore biomass increased significantly more than plant or parasitoid biomass at higher temperature, and this generated an increased ratio of herbivore to plant biomass with warming. Our findings of greatly increased herbivore biomass at higher temperature support hypotheses of increased herbivory based on data from agricultural systems (reviewed by Rustad *et al*. [8], Bale *et al.* [9] and Throop and Lerdau [46]) and paleological records [47], [48].

In contrast to warming, the strength of the nitrogen direct effect on relative biomass change decreased from plants (strong positive response) to herbivores (marginally-significant positive response) to parasitoids (no response), suggesting that bottom-up effects, or increases in resource availability, had decreasing strength or efficiency moving up the food chain. The importance of bottom-up effects was emphasized by the significant effect of plant on herbivore, and herbivore on parasitoid biomass in both experiments, and after controlling for these effects, nitrogen had no significant effect on herbivore or parasitoid biomass. In light of these findings, the role of natural enemies in controlling herbivore populations is likely to be strongly impaired by both drivers (an hypothesis supported by the significant reduction in parasitism rates under elevated temperature or nitrogen).

In addition to biomass, rates of herbivory are also predicted to increase at higher levels of nitrogen availability, which could in turn support larger herbivore populations [49]. Although there may have been a top-down reduction in plant biomass due to elevated herbivory, this was not sufficient to outweigh the effect of nitrogen on plant growth, a finding congruent with that of Throop [46], who showed that positive impacts of N on shoot biomass were typically not significantly suppressed by herbivory. In our experiments, we found that both plants and herbivores substantially gained biomass under elevated nitrogen, indicating a generally more productive system at the plant and herbivore level, but not at the parasitoid level.

Interestingly, under elevated levels of both temperature and nitrogen, we observed a higher increase in biomass of herbivores than parasitoids, whilst the increase of herbivores and plants was qualitatively similar. In other words, the presence of nitrogen as a second driver mitigated the strong difference in response between plants and herbivores at higher temperatures. This result highlights the importance of considering the co-occurrence of global change drivers; under a scenario of global warming with no increase in nitrogen deposition, herbivores show a clearly stronger response than plants and parasitoids. However, under a realistic scenario of co-occurring drivers [3], [5], [16], the difference in response, particularly between plants and herbivores, may be less than expected when considering each driver in isolation.

Contrastingly, we found strong evidence in both field experiments that natural enemies were not able to respond as positively to increased herbivore (host) resource availability under a changing environment. Parasitoid biomass did not significantly increase under any treatment and, importantly, showed a significantly lower response than herbivores at higher temperature. Moreover, both experiments qualitatively showed a net negative effect of temperature on parasitoids. Once we had accounted for the predictable correlation between herbivore biomass and parasitoid biomass, we found a trend for a negative effect of temperature on parasitoid biomass in the artificial warming experiment. Similarly, in the elevation gradient experiment, we found that the biomass correlation between parasitoids and herbivores was weaker at higher temperatures, and led to a decreasing parasitoid-herbivore biomass ratio. These results suggest that parasitoids were not able to counteract the strong response of herbivores (perhaps because parasitoid population responses were too slow), and this effectively generated a situation of predator-release under elevated temperature. This view is also supported by the significantly lower rates of parasitism found in the gradient experiment. Even though parasitoids attacked significantly more hosts under elevated temperature (results not shown), this increase was not proportionate to the increase in host abundance, which generated a lower proportion of hosts parasitised. It must also be noted that parasitism rates did not differ significantly across treatments in the artificial warming experiment. However, due to the small distance between plots, it is plausible that parasitoids could display behavioral choices to attack hosts across different treatments depending on their availability at a given time. Therefore, the parasitism results in the artificial warming experiment should be taken cautiously and should not undermine the validity of the results we obtained under natural field conditions in the elevational gradient study.

Our results contrast with those of Andrew and Hughes [50], who found no evidence for increased ratios of herbivores to parasitoids and other natural enemies along a latitudinal gradient. However, the scope and methodology of their study shows substantial differences from ours. Andrew and Hughes sampled all arthropods by knocking them down from the host plant using a pyrethrum/water solution. They thus obtained data on abundance and biomass of the major insect taxa sorted into feeding groups, but would have also included ‘tourist’ species, which may not have been feeding on the plants or herbivores. In contrast, we reared parasitoids from living hosts, which incorporates host-selection effects on parasitoids, and provides a measure of biomass that directly relates to the ecosystem function of parasitism. Thus, our estimate of biomass is obtained from parasitoids that are not merely present, but also able to interact successfully with their host. Nevertheless, the results of Andrew and Hughes imply that sampling of free-living adult parasitoids could lead to different results.

Strengthened top-down control by generalist predators observed under warming in terrestrial systems [51] suggests that more specialized natural enemies such as parasitoids may be less responsive than generalists. Temperature is known to increase metabolic rates of mobile predators such as spiders [25] whilst, in contrast, parasitoid development is dependent on their host, which may constrain (e.g., through changes in host phenology or quality) their ability to adapt to change.

Previous studies on tri-trophic food chains concluded that parasitoids are unlikely to effectively counteract the response of herbivores to climate change [52], and specifically suggested that bottom-up forces may be more important than top-down control by the parasitoids [53]. Our findings are congruent with this suggestion, and show a severe limitation in the ability of parasitoids to effectively control herbivore populations. Our findings have concerning implications for biological control of herbivore pests, and suggest that herbivores will be the most likely to benefit and thrive in a changing environment.

## Supporting Information

Appendix S1 **Altitudinal gradient location details.** GPS coordinates, altitude and mean temperature of each sampling plot, and photographic map of their location.
(DOC)

Appendix S2 **Artificial warming experiment: study location and experimental temperature control.** Location of the two experiments in the landscape of the South Island of New Zealand, photographic timeline of the artificial warming experiment set up and graphic details of the temperature control system.
(DOC)

Appendix S3 **Biomass ratio analyses.** Coefficient tables for the analyses of parasitoid-herbivore and herbivore-plant biomass ratios
(DOC)
