# Supplementary material for: Climate Change Disproportionately Increases Herbivore over Plant or Parasitoid Biomass
Source: PLoS One. 2012 Jul 18;7(7):e40557. doi: 10.1371/journal.pone.0040557 (PMC3399892; doi:10.1371/journal.pone.0040557)
Supplement: Appendix S1 — Altitudinal gradient location details. GPS coordinates, altitude and mean temperature of each sampling plot, and photographic map of their location. (DOC) [file pone.0040557.s001.doc]

**Appendix S1: Altitudinal gradient location details**

**Table 1: Sampling location details**. Sampling was conducted along 5 transects, each with 3 different elevations (plots) and a nitrogen subplot at each elevation point. Subplots are not separated here, as values apply to the entire plot. Coordinates are based on a GPS New Zealand map with EGS84 reference grid.

| **Transect** | **Elevation** | **Altitude (m.a.s.l)** | **Coordinates**  **North (N)** | **East (E)** | **Mean**  **Temperature (°C)** |
| --- | --- | --- | --- | --- | --- |
| **DE** | Bottom | 650 | N 584 3746 | E 246 5971 | 6.72 |
|  | Mid | 790 | N 584 3468 | E 246 5740 | 5.61 |
|  | Top | 940 | N 584 3367 | E 246 5739 | 5.00 |
| **KE** | Bottom | 732 | N 583 9796 | E 245 8308 | 5.31 |
|  | Mid | 891 | N 583 9287 | E 245 8289 | 5.79 |
|  | Top | 1031 | N 583 9021 | E 245 8168 | 5.03 |
| **DW** | Bottom | 724 | N 584 3754 | E 246 4325 | 6.55 |
|  | Mid | 880 | N 584 3364 | E 246 4341 | 6.00 |
|  | Top | 1009 | N 584 3109 | E 246 4357 | 4.49 |
| **NS** | Bottom | 743 | N 584 0489 | E 245 8540 | 5.64 |
|  | Mid | 883 | N 584 0796 | E 245 8657 | 5.08 |
|  | Top | 1050 | N 584 1071 | E 245 8817 | 4.03 |
| **LZ** | Bottom | 792 | N 5840276 | E2456733 | 5.29 |
|  | Mid | 937 | N 5840655 | E 2456562 | 4.79 |
|  | Top | 1073 | N 5840791 | E 2456751 | 3.89 |

**Figure 1:** Satellite imagery of the experiment area of the Hope River, South Island, New Zealand (Source: Google Earth), showing locations of the transects (white lines) and their code (See Table 1 for topographic details and coordinates).
