# Supplementary material for: Climate Change Disproportionately Increases Herbivore over Plant or Parasitoid Biomass
Source: PLoS One. 2012 Jul 18;7(7):e40557. doi: 10.1371/journal.pone.0040557 (PMC3399892; doi:10.1371/journal.pone.0040557)
Supplement: Appendix S2 — Artificial warming experiment: study location and experimental temperature control. Location of the two experiments in the landscape of the South Island of New Zealand, photographic timeline of the artificial warming experiment set up and graphic details of the temperature control system. (DOC) [file pone.0040557.s002.doc]

**Appendix S2: Artificial warming experiment: study location and experimental temperature control**


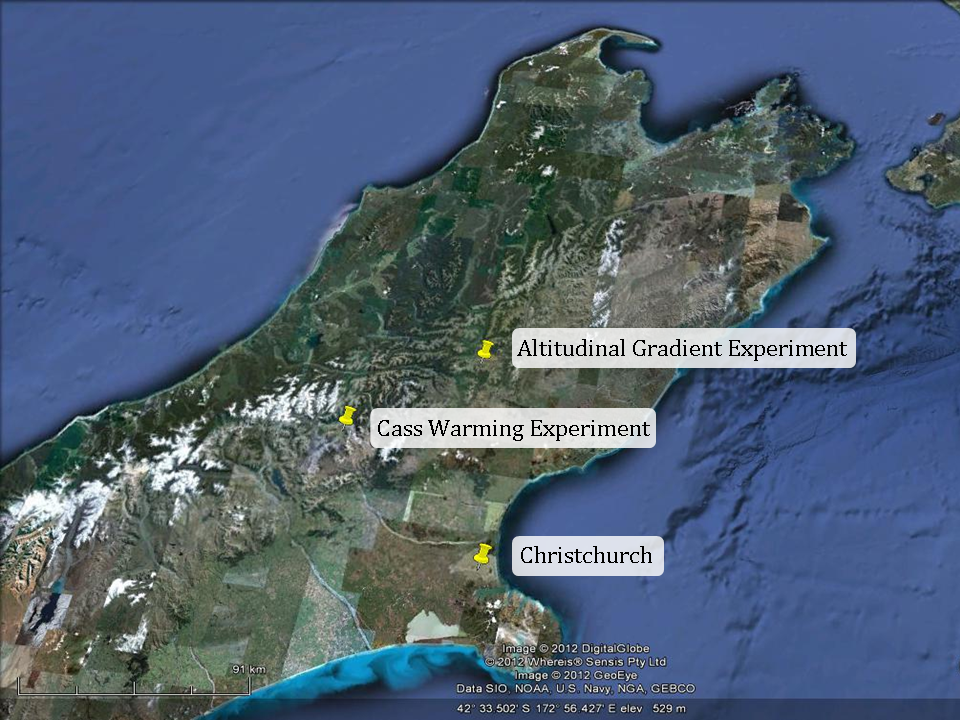


**Figure 2:** Satellite imagery of the upper half of the South Island, New Zealand (Source: Google Earth), showing the location of the artificial warming experiment at Cass, in the southern alps, in relation to the city of Christchurch.


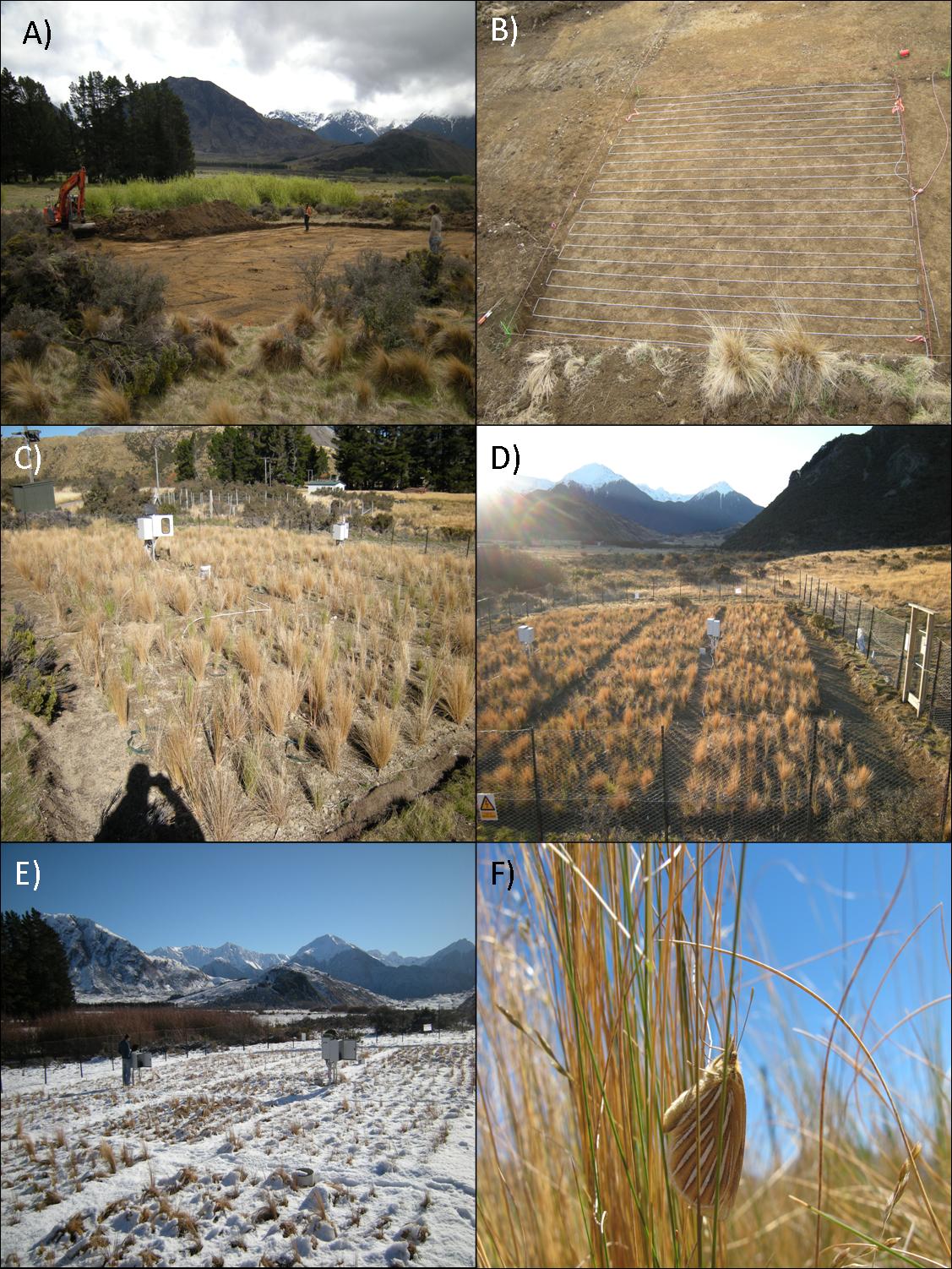


**Figure 3**: Photo sequence of the establishment of the Cass warming experiment. A) We dug the experimental area in October 2008, B) installed the heating cables in each plot with standardized layout, buried them in the ground and C) planted 2880 tussocks, 144 per plot with replicated composition. D) The experimental warming was firstl activated in April 2009 and the experiment was let run E) through winter and spring, before F) we began sampling Lepidoptera larvae in January (midsummer) 2010.

**Figure 4**: Average monthly temperature (±SE) for Aug 2009-July 2010 in a heated plot against the control plot and differential temperature between the treatments. Monthly averages are calculated as the average daily temperature for that month. The experimental warming maintained the temperature difference between heated and control plots very close to the three degree target throughout the year.

**Figure 5**: Average daily temperature for October 2010 (the month of overall peak larval abundance) in a heated plot against the control plot and differential temperature between the treatments. For the heating plot, an average (±SE) is calculated from the three thermocouples in the plot. For the control plot, values are the daily average of the one thermocouple installed. The experimental warming maintained the temperature difference between heated and control plots very close to the three degree target, and accurately followed daily temperature fluctuations.
