# Supplementary material for: Climate Change Disproportionately Increases Herbivore over Plant or Parasitoid Biomass
Source: PLoS One. 2012 Jul 18;7(7):e40557. doi: 10.1371/journal.pone.0040557 (PMC3399892; doi:10.1371/journal.pone.0040557)
Supplement: Appendix S3 — Biomass ratio analyses. Coefficient tables for the analyses of parasitoid-herbivore and herbivore-plant biomass ratios (DOC) [file pone.0040557.s003.doc]

**Appendix S3: biomass ratio analyses**

Table 1: Table coefficient for the biomass ratio analyses

A) Altitudinal gradient experiment: herbivore to plant biomass ratio

Value Std.Error DF t-value p-value

(Intercept) -14.70 1.74 13 -8.44 <0.0001

Temperature 1.41 0.32 9 4.32 0.0019 **

Nitrogen 2.08 1.58 13 1.32 0.2087

Temp:nitrogen -0.35 0.29 13 -1.17 0.2614

B) Altitudinal gradient experiment: parasitoid to herbivore biomass ratio

Value Std.Error DF t-value p-value

(Intercept) -1.97 1.30 13 -1.51 0.154

Temperature -0.40 0.24 9 -1.91 0.084 .

Nitrogen -1.68 1.22 13 -1.39 0.188

Temp:Nitrogen 0.25 0.23 13 1.09 0.295

C) Artificial warming experiment: herbivore to plant biomass ratio

Estimate Std.Error t value Pr(>|t|)

(Intercept) -6.18 0.12 -52.42 <0.0001

Warming 0.42 0.17 2.50 0.024 *

Nitrogen -0.02 0.17 -0.14 0.888

Warmingw:nitrogen -0.27 0.24 -1.16 0.264

D) Artificial warming experiment: parasitoid to herbivore biomass ratio

Estimate Std.Error t value Pr(>|t|)

(Intercept) -3.78 0.24 -15.88 <0.0001***

Warmingw -0.60 0.34 -1.78 0.094 .

Nitrogenn -0.40 0.34 -1.19 0.251

Warmingw:nitrogen 0.65 0.48 1.37 0.190
